# Supplementary material for: Structural and immunological characterization of the H3 influenza hemagglutinin during antigenic drift
Source: Nat Commun. 2025 Dec 11;16:11452. doi: 10.1038/s41467-025-66375-7 (PMC12749422; doi:10.1038/s41467-025-66375-7)
Supplement: Supplementary file 1 — Supplementary Information [file 41467_2025_66375_MOESM1_ESM.pdf]

Structural and immunological characterization of the H3 influenza hemagglutinin during antigenic drift

Rebeca de Paiva Froes Rocha[1,2,8], Ilhan Tomris[3], Charles A. Bowman[1], Emma Stevens[4,5], Jason Kantorow[4,5], Corinna M. Plitt[6], Weiwei Peng[6], Svearike Oeverdieck[1], Thales Galdino Andrade[1], James A. Ferguson[1], Diana D. Jung[1], Rafael Elias Marques[2], Sander Herfst[7], Joost Snijder[6], Srirupa Chakraborty[4,5], Alba Torrents de la Peña[1], Zachary T. Berndsen[1,9,*], Robert P. de Vries[3,*], Andrew B. Ward[1,10,*]

[1] Department of Integrative Structural and Computational Biology, The Scripps Research Institute, La Jolla, CA 92037, USA.
[2] Brazilian Biosciences National Laboratory (LNBio), Brazilian Center for Research in Energy and Materials (CNPEM), Campinas, 13083-970, Brazil.
[3] Department of Chemical Biology and Drug Discovery, Utrecht Institute for Pharmaceutical Sciences, Utrecht University, Utrecht, The Netherlands.
[4] Department of Chemical Engineering, Northeastern University, Boston, MA 02115, USA.
[5] Department of Chemistry and Chemical Biology, Northeastern University, Boston, MA 02115, USA.
[6] Biomolecular Mass Spectrometry and Proteomics, Bijvoet Center for Biomolecular Research, Utrecht Institute of Pharmaceutical Sciences, Utrecht University, Utrecht, The Netherlands
[7] Department of Viroscience, Erasmus Medical Center, Rotterdam, The Netherlands
[8] Current address: Department of Surgery, University of California San Francisco School of Medicine, San Francisco, CA 94143, USA
[9] Current address: Department of Molecular and Cell Biology, University of California, Berkeley, CA, USA.
[10] Lead contact: andrew@scripps.edu

* Corresponding authors: Zachary T. Berndsen (zberndsen@missouri.edu), Robert P. de Vries (r.vries@uu.nl) and Andrew B. Ward (andrew@scripps.edu)

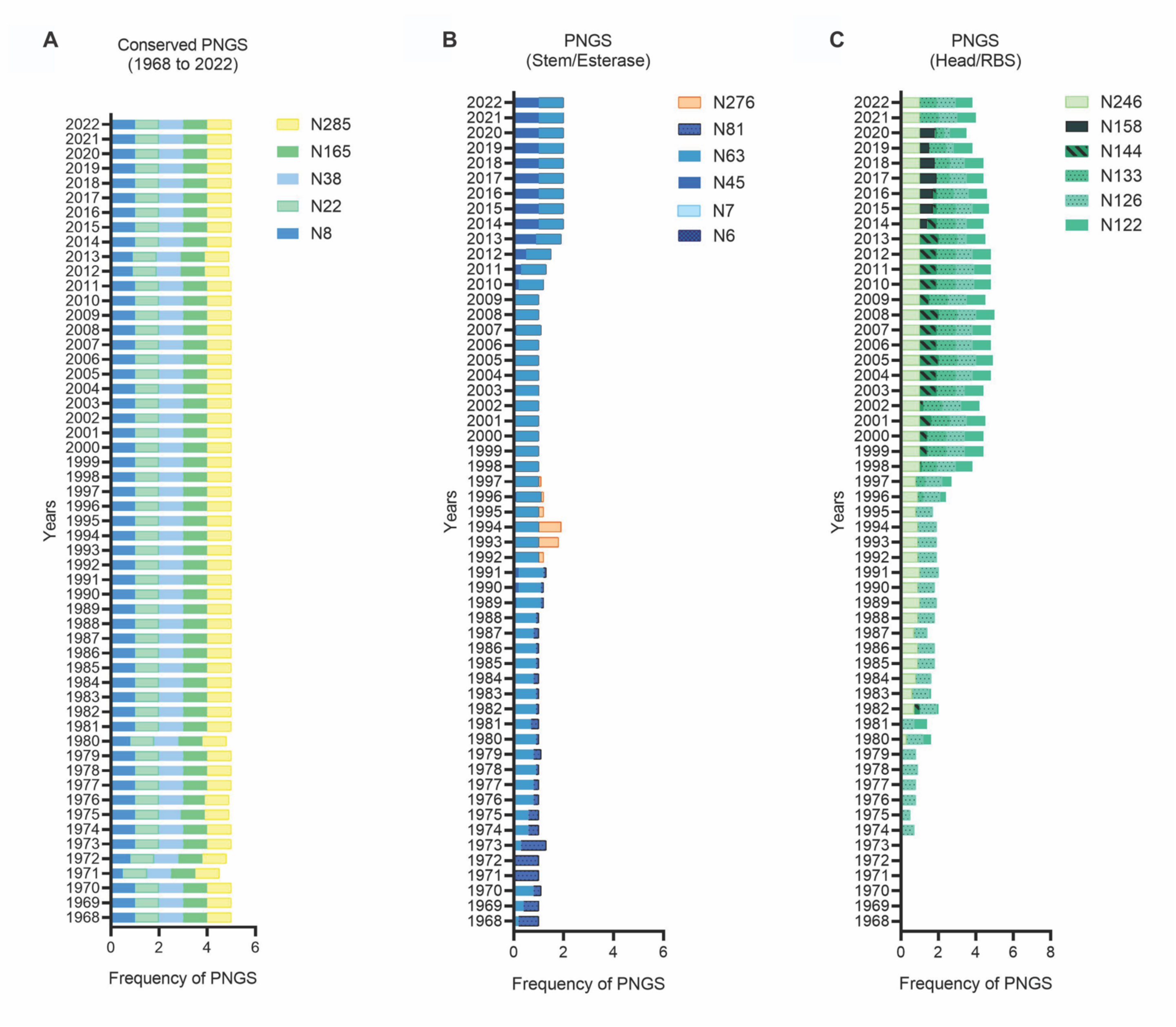

Supplementary Figure 1. Evolution of PNGS distribution in different regions of HA over time. (**A**) Glycans that are consistently present on H3 circulating strains from 1968 to 2022. (**B**) Glycans present on stem and esterase regions and (**C**) glycans present on head and RBS regions of H3 circulating strains from 1968 to 2022. This analysis includes ~11000 sequences, which were downloaded from the influenza research database.[22] Source data are provided as a Source Data file.

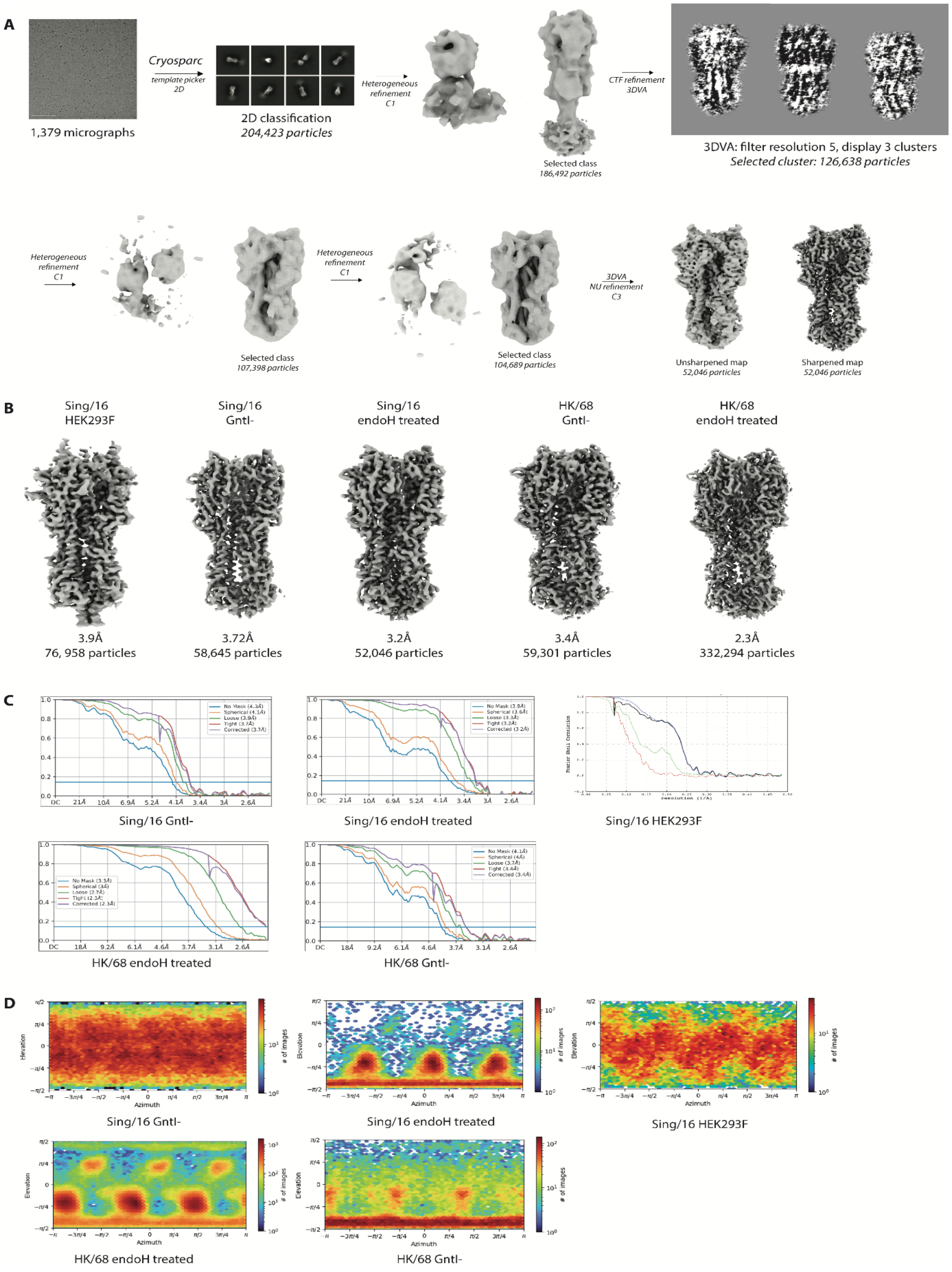

Supplementary Figure 2. Cryo-EM processing workflow. (**A**) Schematic representation of cryo-EM data processing workflow for endoH-treated Sing/16. The rest of the structures were processed similarly. 3DVA: 3D Variability Analysis. NU: non-uniform refinement. (**B**) Individual cryo-EM maps. (**C**) Fourier shell correlation (FSC) plots corresponding the reconstructions shown in panel B. While HK/68, Sing/16 GntI-, endo-H treated Sing/16 were processed using CryoSparc, Sing/16 HEK293F was processed using relion. The color code for the FSC of Sing/16 HEK293F is the following: no mask is orange, spherical mask is green, corrected mask is black and tight mask is purple. (**D**) Angular distribution calculated using CryoSparc for all 5 H3 HAs.

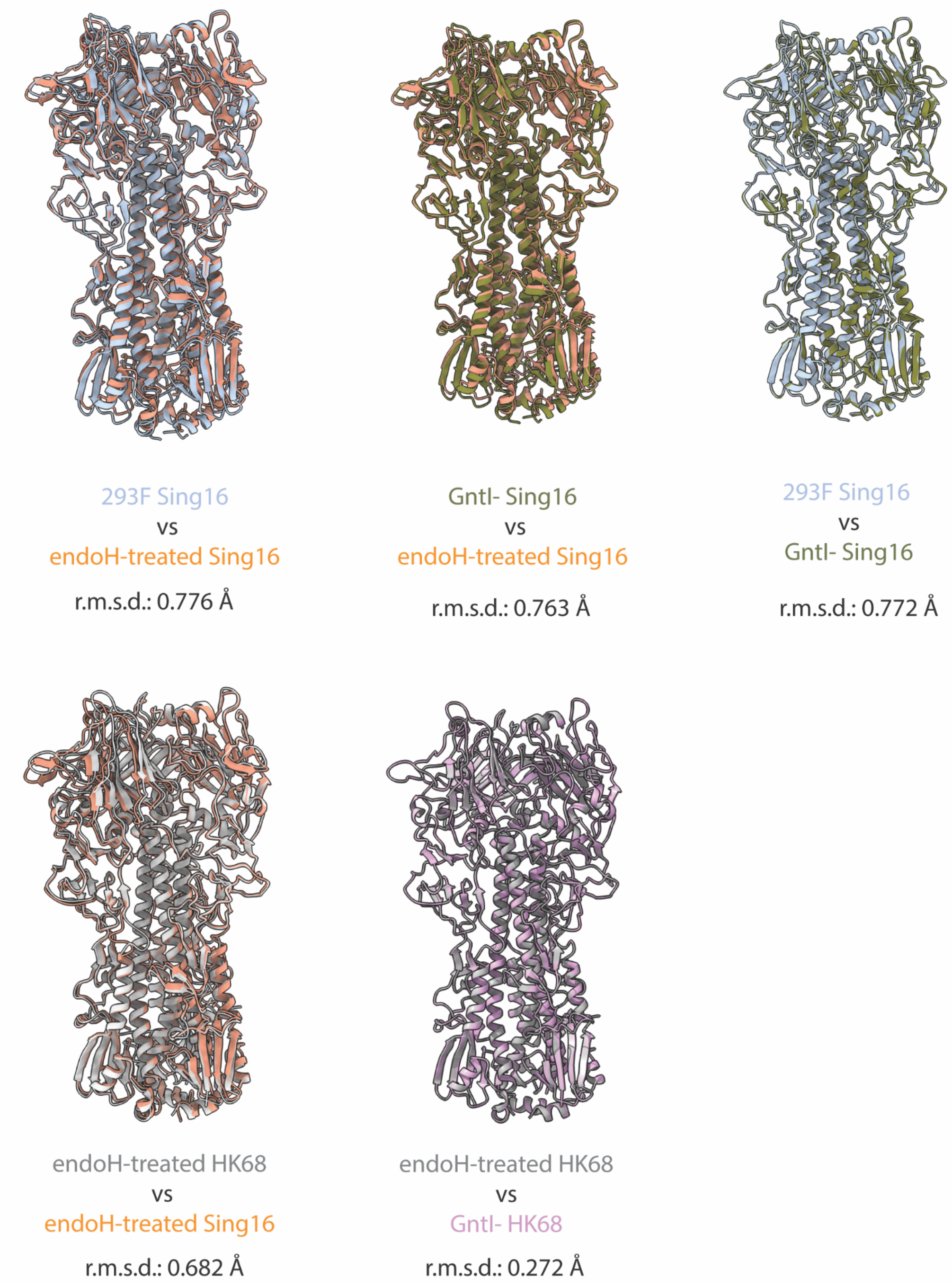

Supplementary Figure 3. Overlay of cryo-EM models of Sing/16 and HK/68 expressed in different cell lines and after Endo H treated. Cryo-EM models were overlayed and pairwise r.m.s.d. (Å) was performed with one point per residue using ChimeraX. r.m.s.d.: root mean square deviation.

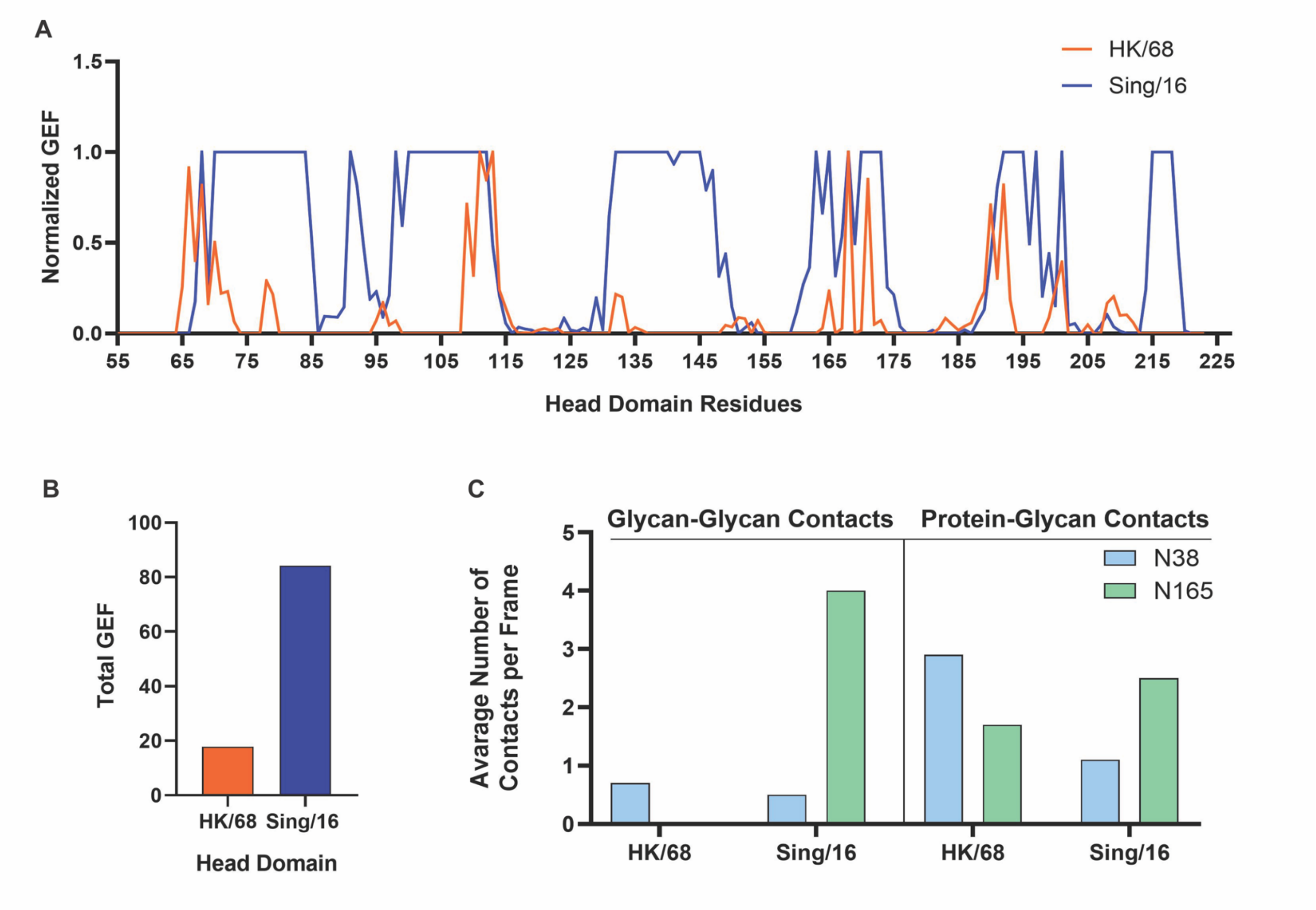

Supplementary Figure 4. Glycan Shield Analysis. **(A)** Residue-wise average normalized glycan encounter factor (GEF), which is a quantification of the glycan shielding effect over the HA head domain residues of C52 to C277. HK/68 residues shown in red, and Sing/16 in blue. **(B)** Sum of normalized GEF over all residues of the HA head domain **(C)** Glycan-Glycan and Glycan-Peptide contacts on the HA head for HK/68 and Sing/16. Source data are provided as a Source Data file.

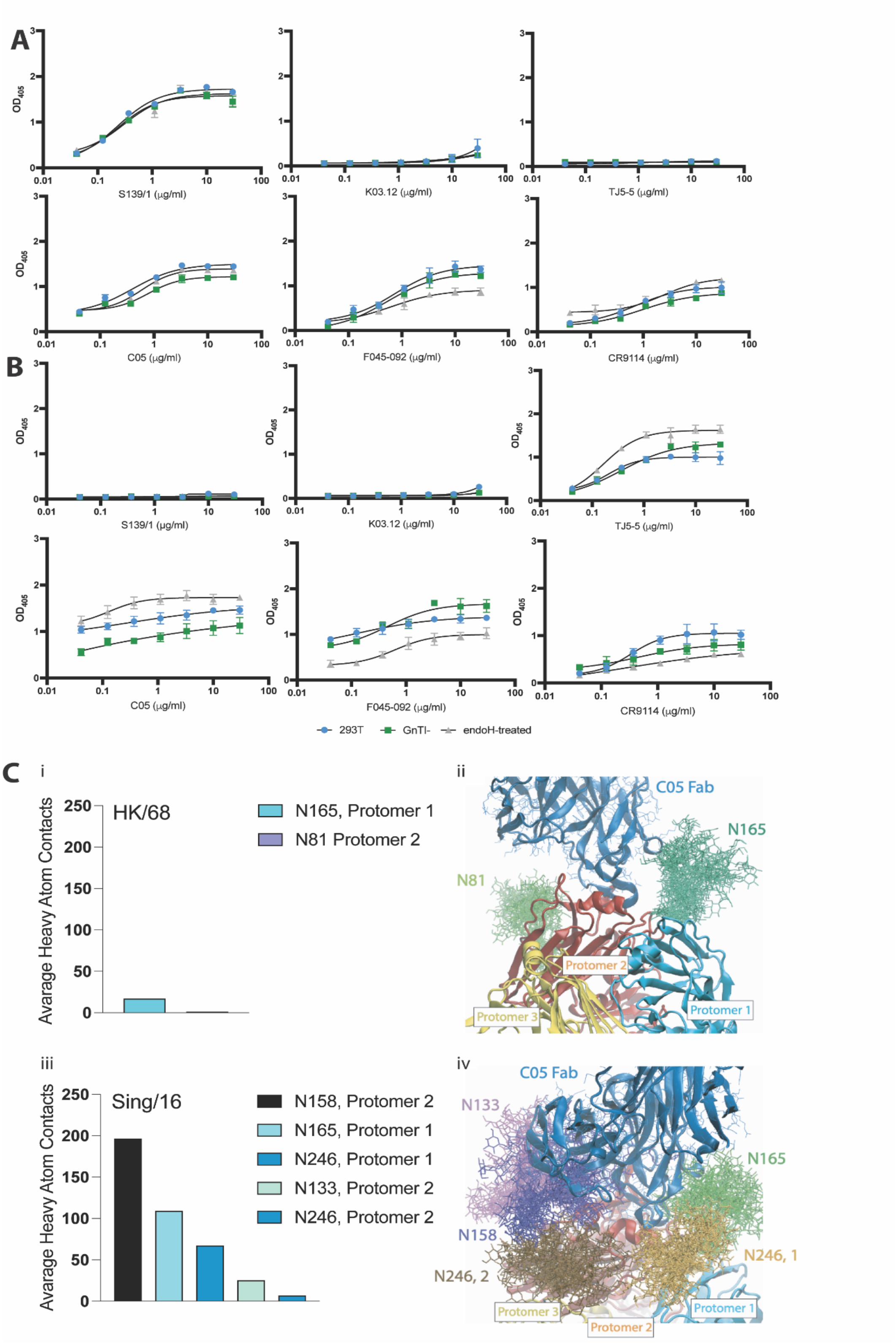

Supplementary Figure 5. Binding of bNAbs to RBS and stem of HK/68 and Sing/16. ELISA binding curves for bNAbs targeting the head and stem of HK/68 (**A**) and Sing/16 (**B**) produced in HEK 293T (blue), GnTI- (green) and endoH treated HEK293T (gray). Experiments were performed in duplicate and error bars are shown. OD_{405}: optical density at 405nm. (**C**) Average number of heavy atom (non-hydrogen) contacts between C05 Fab and the surrounding N-linked glycans when bound to HK/68 and Sing/16 (I,III) calculated from the computationally generated ensembles (II, IV). The threshold value used to denote a contact for both analyses was set at 4.5Å. Source data are provided as a Source Data file.

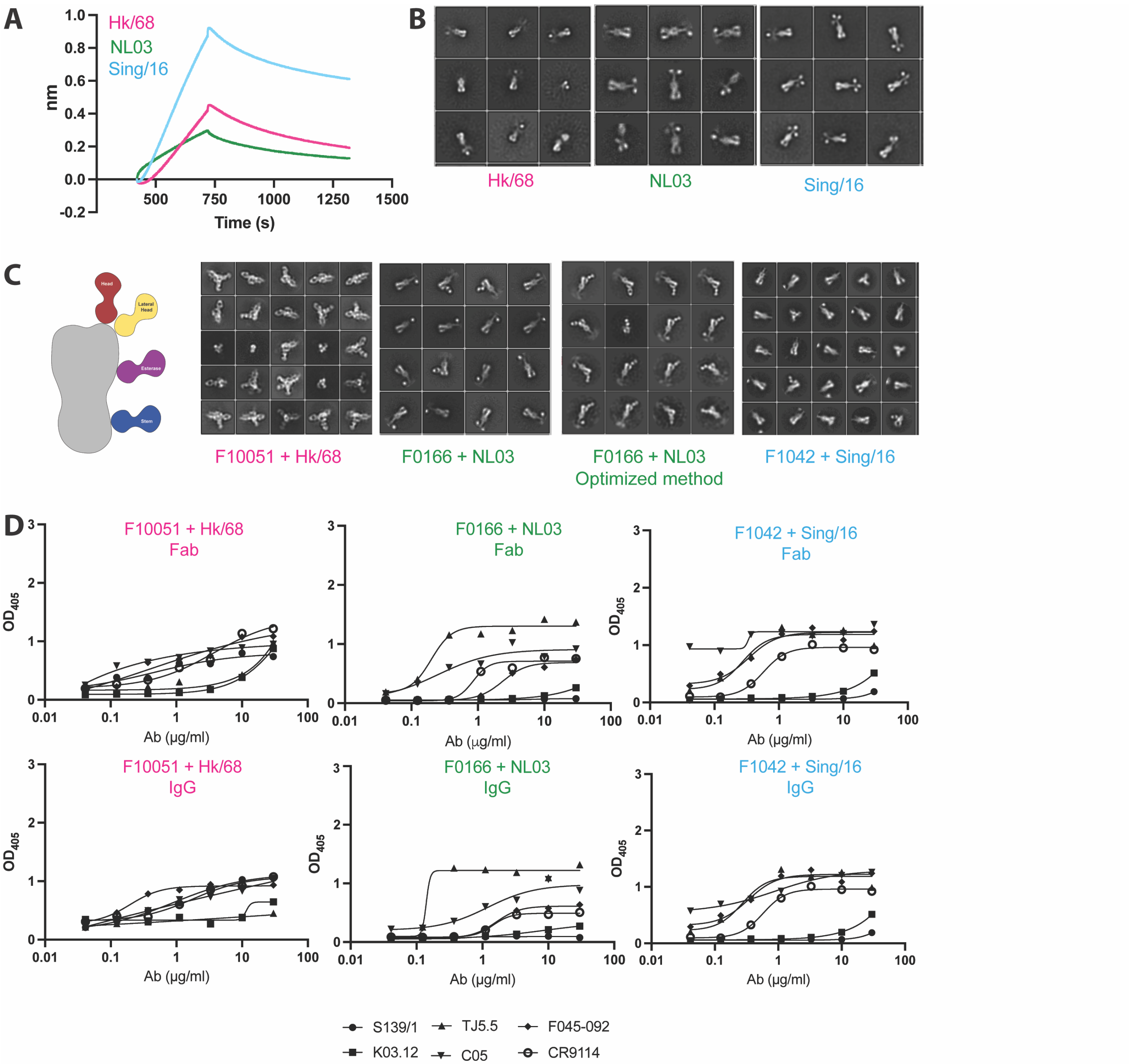

Supplementary Figure 6. Binding of polyclonal antibodies from reference sera to matching protein. (**A**) Binding of ferret sera with the matching hemagglutinin was performed using BLI. (**B**) 2D classification of apo HA trimers HK/68, NL/03 and Sing/16. (**C**) 2D classification from EMPEM analysis of polyclonal Fabs obtained from reference sera (F10051, F0166 and F10421) complexed with matching protein (HK/68, NL/03 and Sing/16) purified from HEK 293T cells. (**D)** Competition ELISA using Fab or IgG as competitors at a concentration of 20µg/µl. Source data are provided as a Source Data file.

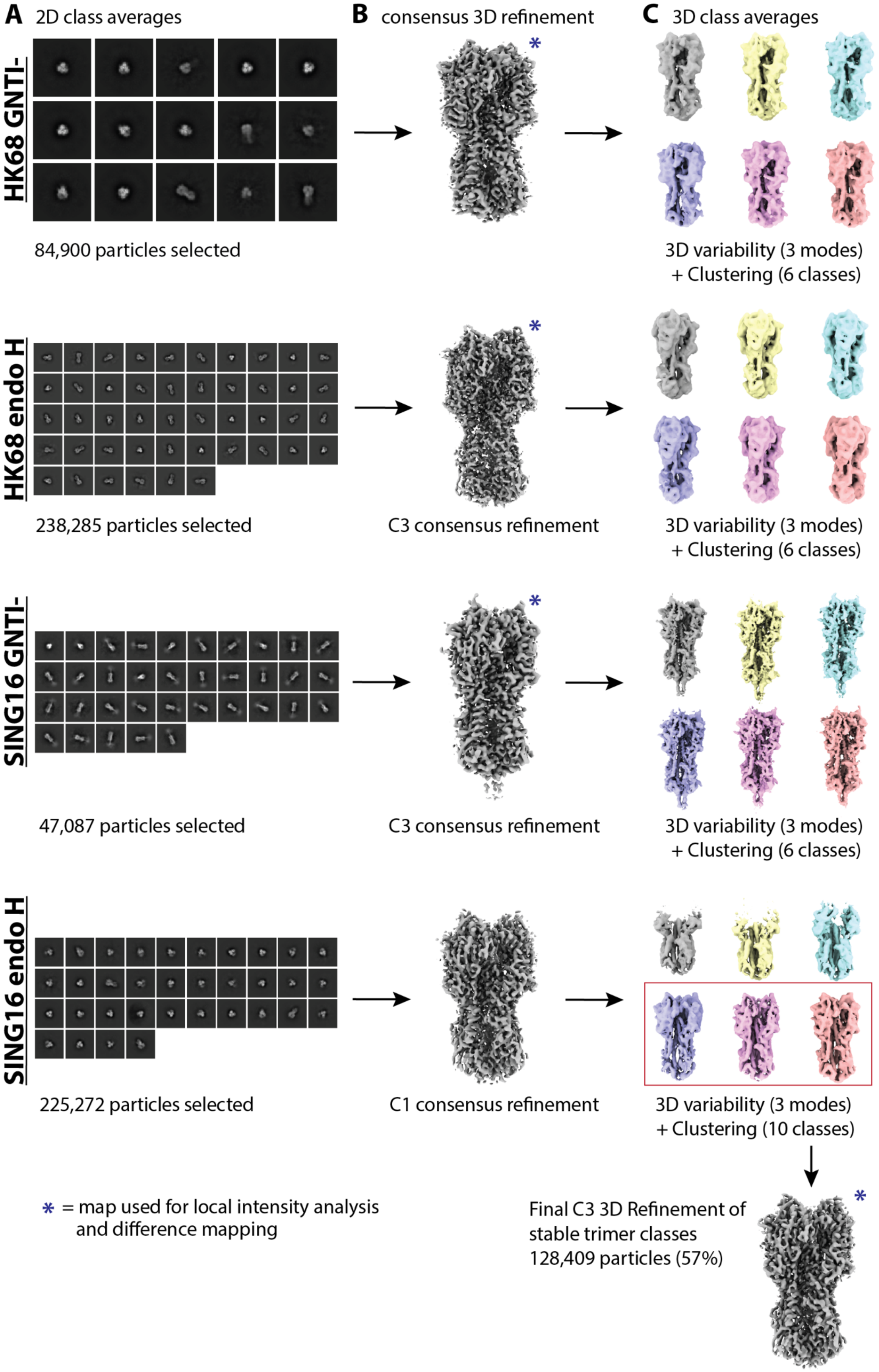

Supplementary Figure 7. Processing pipeline for discriminating stable and non-intact HA trimers.

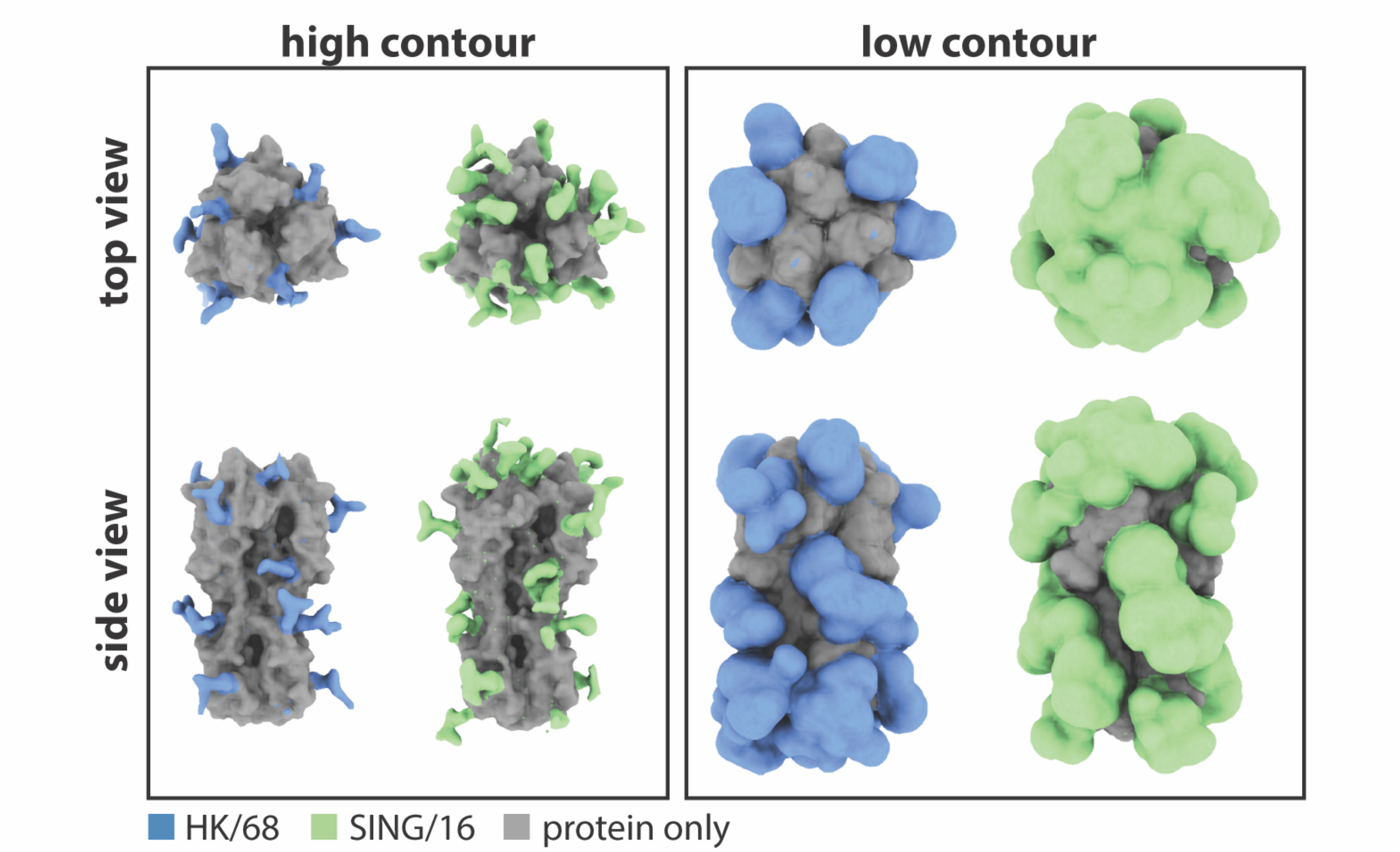

Supplementary Figure 8. Simulated cryo-EM maps. Synthetic cryo-EM density maps generated from the 1000 atomistic models (Figure 3) of fully glycosylated HK/68 (blue) and SING/16 (green) HAs and from protein residues only (grey) shown at high and low contour levels from the top and side.

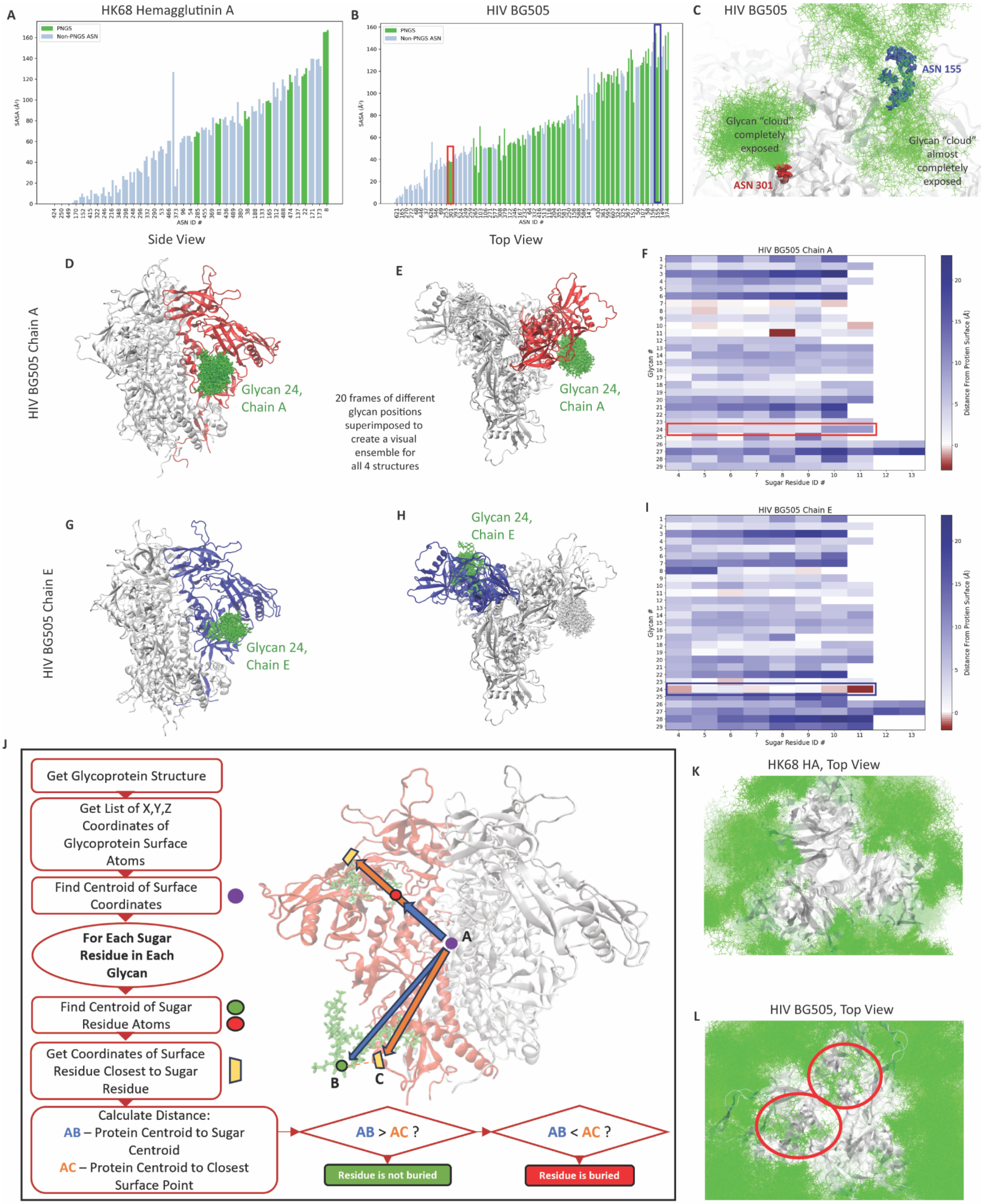

Supplementary Figure 9. An overview of example outputs of the SASA and Centroid methods of finding buried glycans within viral glycoproteins. Example plots of SASA vs. the residue identification number of

each asparagine in the case of Human Influenza HK68 hemagglutinin A **(A)** and HIV BG505 **(B)** are shown with highlights of the least and greatest SASA PNGS residues shown in red and blue, respectively. A graphical representation of the two SASA extremes of HIV BG505 is shown in **(C)**, where both highlighted residues have similar degrees of glycan exposure regardless of their SASA values. Heat map outputs of the succeeding centroid method in **(F)** and **(I)** depicting the depths below or distance above the protein scaffold surface for each sugar residue of each glycan of an average over 100 frames of an HIV structural trajectory emphasize glycan 24 to demonstrate the visual contrast created between an unburied and buried glycan, respectively. This can be visually seen between the contrast of side and top views of the unburied glycan 24 on HIV BG505 chain A in **(D)** and **(E)**, and the buried glycan 24 on HIV BG505 chain E in **(G)** and **(H)**. The decision tree and graphical depiction in **(J)** shows the logic behind the centroidal method of identifying buried glycans. Finally, two top-down renders of 50 frames of all glycans of **(K)** (Human Influenza HK68 Hemagglutinin A) and **(L)** (HIV) display the contrast between the degree of glycan burial between a structure with almost no glycan burial and one with a high degree of glycan burial highlighted in orange, respectively. Source data are provided as a Source Data file.

Supplementary Table 1. Number and percentage of circulating strains containing NXT/S motif at certain amino acid positions

Supplementary Table 1.																		
**Position**	6	7	8	22	38	45	63	81	122	126	133	144	158	165	246	276	483	Total of circulating strains
**Year**	Number of circulating strains																	
1968	0	0	22	22	22	0	5	17	0	1	0	0	0	22	0	0	21	22
1969	0	0	5	5	5	0	2	3	0	0	0	0	0	5	0	0	5	5
1970	0	0	4	4	4	0	3	1	0	0	0	0	0	4	0	0	4	4
1971	0	0	4	8	8	0	0	8	0	0	0	0	0	8	0	0	8	8
1972	0	0	12	15	15	0	0	15	0	0	0	0	0	15	0	0	15	15
1973	0	0	7	7	7	0	2	7	0	0	0	0	0	7	0	0	7	7
1974	0	0	10	10	10	0	6	4	0	7	0	0	0	10	0	0	10	10
1975	0	0	10	10	9	0	6	4	0	5	0	0	0	10	0	0	10	10
1976	0	0	12	12	12	0	10	2	0	10	0	0	0	11	0	0	12	12
1977	0	0	11	11	11	0	9	2	0	9	0	0	0	11	0	0	11	11
1978	0	0	7	7	7	0	6	1	0	6	0	0	0	7	0	0	7	7
1979	0	0	4	4	4	0	3	1	0	3	0	0	0	4	0	0	4	4
1980	0	0	6	8	8	0	7	1	3	7	0	0	0	8	2	0	8	8
1981	0	0	3	3	3	0	2	1	2	2	0	0	0	3	0	0	3	3
1982	0	0	10	10	10	0	9	1	1	9	0	3	0	10	7	0	10	10
1983	0	0	7	7	7	0	6	1	1	6	0	0	0	7	4	0	7	7
1984	0	0	5	5	5	0	4	1	0	4	0	0	0	5	4	0	5	5
1985	0	0	14	14	14	0	13	1	0	13	0	0	0	14	13	0	14	14
1986	0	0	8	8	8	0	7	1	0	7	0	0	0	8	7	0	8	8
1987	0	0	6	6	6	0	5	1	0	4	0	0	0	6	4	0	6	6
1988	0	0	11	11	11	0	10	1	0	10	0	0	0	11	10	0	11	11
1989	0	0	20	20	20	1	19	1	0	18	0	0	0	20	19	0	20	20
1990	0	0	9	9	9	2	8	1	0	8	0	0	0	9	8	0	9	9
1991	0	0	20	20	20	3	19	1	0	19	0	0	0	20	19	0	20	20
1992	0	0	21	21	21	0	20	1	0	20	0	0	0	21	19	4	21	21
1993	0	0	68	68	68	0	67	1	0	67	0	0	0	68	59	54	68	68
1994	0	0	45	45	45	0	44	1	0	44	0	0	0	45	39	42	45	45

1995	0	0	46	46	46	0	45	1	0	43	0	0	0	45	38	10	46	46
1996	0	0	56	55	56	3	55	1	18	53	18	0	0	56	50	3	56	56
1997	0	0	38	39	39	1	38	1	18	37	20	0	0	38	33	3	39	39
1998	1	0	43	44	44	0	43	1	38	43	34	6	0	44	43	0	44	44
1999	0	0	111	112	111	3	111	1	108	111	111	42	0	109	108	0	112	112
2000	0	0	85	86	86	0	85	1	85	85	85	37	0	83	84	0	86	86
2001	0	0	50	51	51	2	50	1	49	48	50	29	0	50	50	0	51	51
2002	0	0	110	115	115	4	114	1	114	113	114	22	0	112	114	0	115	115
2003	0	1	184	187	187	4	186	1	182	89	186	172	0	182	185	0	187	187
2004	0	0	149	152	152	2	150	1	148	130	151	140	0	150	148	0	152	152
2005	0	0	151	154	154	3	153	1	145	151	152	148	0	152	150	0	154	154
2006	0	0	85	85	85	1	82	1	82	80	84	80	0	85	84	0	85	85
2007	1	1	228	232	232	25	231	1	222	218	229	212	0	229	230	0	232	232
2008	0	0	169	171	171	1	169	1	166	166	170	168	0	170	169	0	171	171
2009	0	0	296	301	299	5	299	1	293	297	289	140	0	298	297	0	301	301
2010	1	0	301	310	309	54	309	1	292	306	299	275	0	309	306	0	291	310
2011	1	0	432	452	452	123	449	1	426	447	434	409	0	450	444	0	451	452
2012	1	0	595	641	643	347	640	1	621	569	636	598	0	639	628	0	641	643
2013	2	1	486	514	517	478	515	1	499	261	513	495	4	516	512	0	517	517
2014	1	1	634	644	643	636	643	1	550	376	632	310	271	643	636	0	641	644
2015	0	2	865	872	877	869	873	1	798	759	861	143	613	875	863	0	876	877
2016	2	1	819	846	850	838	848	1	815	703	798	135	606	849	843	0	850	850

2017	0	2	1308	1317	1319	1311	1313	1	1203	1230	948	15	1135	1315	1304	0	1316	1319
2018	0	7	841	855	856	849	854	3	830	672	694	3	677	853	855	0	857	857
2019	0	1	810	830	834	828	831	1	812	319	722	5	383	833	833	0	832	835
2020	0	1	93	92	93	91	92	1	85	31	39	11	73	93	91	0	93	93
2021	0	0	167	167	169	167	166	1	164	163	162	0	3	169	167	0	169	169
2022	1	9	1244	1254	1258	1254	1253	1	1195	1247	1176	6	4	1254	1257	0	1259	1260

**Number**	1	2	3	4	5	6	7	8	9	10	11	12	13	14	15	16	17	Total of circulating strains
**Position**	6	7	8	22	38	45	63	81	122	126	133	144	158	165	246	276	483	
**Year**	PNGS (%)																	
1968	0.0	0.0	1.0	1.0	1.0	0.0	0.2	0.8	0.0	0.0	0.0	0.0	0.0	1.0	0.0	0.0	1.0	22
1969	0.0	0.0	1.0	1.0	1.0	0.0	0.4	0.6	0.0	0.0	0.0	0.0	0.0	1.0	0.0	0.0	1.0	5
1970	0.0	0.0	1.0	1.0	1.0	0.0	0.8	0.3	0.0	0.0	0.0	0.0	0.0	1.0	0.0	0.0	1.0	4
1971	0.0	0.0	0.5	1.0	1.0	0.0	0.0	1.0	0.0	0.0	0.0	0.0	0.0	1.0	0.0	0.0	1.0	8
1972	0.0	0.0	0.8	1.0	1.0	0.0	0.0	1.0	0.0	0.0	0.0	0.0	0.0	1.0	0.0	0.0	1.0	15
1973	0.0	0.0	1.0	1.0	1.0	0.0	0.3	1.0	0.0	0.0	0.0	0.0	0.0	1.0	0.0	0.0	1.0	7
1974	0.0	0.0	1.0	1.0	1.0	0.0	0.6	0.4	0.0	0.7	0.0	0.0	0.0	1.0	0.0	0.0	1.0	10
1975	0.0	0.0	1.0	1.0	0.9	0.0	0.6	0.4	0.0	0.5	0.0	0.0	0.0	1.0	0.0	0.0	1.0	10
1976	0.0	0.0	1.0	1.0	1.0	0.0	0.8	0.2	0.0	0.8	0.0	0.0	0.0	0.9	0.0	0.0	1.0	12
1977	0.0	0.0	1.0	1.0	1.0	0.0	0.8	0.2	0.0	0.8	0.0	0.0	0.0	1.0	0.0	0.0	1.0	11
1978	0.0	0.0	1.0	1.0	1.0	0.0	0.9	0.1	0.0	0.9	0.0	0.0	0.0	1.0	0.0	0.0	1.0	7
1979	0.0	0.0	1.0	1.0	1.0	0.0	0.8	0.3	0.0	0.8	0.0	0.0	0.0	1.0	0.0	0.0	1.0	4
1980	0.0	0.0	0.8	1.0	1.0	0.0	0.9	0.1	0.4	0.9	0.0	0.0	0.0	1.0	0.3	0.0	1.0	8
1981	0.0	0.0	1.0	1.0	1.0	0.0	0.7	0.3	0.7	0.7	0.0	0.0	0.0	1.0	0.0	0.0	1.0	3
1982	0.0	0.0	1.0	1.0	1.0	0.0	0.9	0.1	0.1	0.9	0.0	0.3	0.0	1.0	0.7	0.0	1.0	10
1983	0.0	0.0	1.0	1.0	1.0	0.0	0.9	0.1	0.1	0.9	0.0	0.0	0.0	1.0	0.6	0.0	1.0	7
1984	0.0	0.0	1.0	1.0	1.0	0.0	0.8	0.2	0.0	0.8	0.0	0.0	0.0	1.0	0.8	0.0	1.0	5
1985	0.0	0.0	1.0	1.0	1.0	0.0	0.9	0.1	0.0	0.9	0.0	0.0	0.0	1.0	0.9	0.0	1.0	14

1986	0.0	0.0	1.0	1.0	1.0	0.0	0.9	0.1	0.0	0.9	0.0	0.0	0.0	1.0	0.9	0.0	1.0	8
1987	0.0	0.0	1.0	1.0	1.0	0.0	0.8	0.2	0.0	0.7	0.0	0.0	0.0	1.0	0.7	0.0	1.0	6
1988	0.0	0.0	1.0	1.0	1.0	0.0	0.9	0.1	0.0	0.9	0.0	0.0	0.0	1.0	0.9	0.0	1.0	11
1989	0.0	0.0	1.0	1.0	1.0	0.1	1.0	0.1	0.0	0.9	0.0	0.0	0.0	1.0	1.0	0.0	1.0	20
1990	0.0	0.0	1.0	1.0	1.0	0.2	0.9	0.1	0.0	0.9	0.0	0.0	0.0	1.0	0.9	0.0	1.0	9
1991	0.0	0.0	1.0	1.0	1.0	0.2	1.0	0.1	0.0	1.0	0.0	0.0	0.0	1.0	1.0	0.0	1.0	20
1992	0.0	0.0	1.0	1.0	1.0	0.0	1.0	0.0	0.0	1.0	0.0	0.0	0.0	1.0	0.9	0.2	1.0	21
1993	0.0	0.0	1.0	1.0	1.0	0.0	1.0	0.0	0.0	1.0	0.0	0.0	0.0	1.0	0.9	0.8	1.0	68
1994	0.0	0.0	1.0	1.0	1.0	0.0	1.0	0.0	0.0	1.0	0.0	0.0	0.0	1.0	0.9	0.9	1.0	45
1995	0.0	0.0	1.0	1.0	1.0	0.0	1.0	0.0	0.0	0.9	0.0	0.0	0.0	1.0	0.8	0.2	1.0	46
1996	0.0	0.0	1.0	1.0	1.0	0.1	1.0	0.0	0.3	0.9	0.3	0.0	0.0	1.0	0.9	0.1	1.0	56
1997	0.0	0.0	1.0	1.0	1.0	0.0	1.0	0.0	0.5	0.9	0.5	0.0	0.0	1.0	0.8	0.1	1.0	39
1998	0.0	0.0	1.0	1.0	1.0	0.0	1.0	0.0	0.9	1.0	0.8	0.1	0.0	1.0	1.0	0.0	1.0	44
1999	0.0	0.0	1.0	1.0	1.0	0.0	1.0	0.0	1.0	1.0	1.0	0.4	0.0	1.0	1.0	0.0	1.0	112
2000	0.0	0.0	1.0	1.0	1.0	0.0	1.0	0.0	1.0	1.0	1.0	0.4	0.0	1.0	1.0	0.0	1.0	86
2001	0.0	0.0	1.0	1.0	1.0	0.0	1.0	0.0	1.0	0.9	1.0	0.6	0.0	1.0	1.0	0.0	1.0	51
2002	0.0	0.0	1.0	1.0	1.0	0.0	1.0	0.0	1.0	1.0	1.0	0.2	0.0	1.0	1.0	0.0	1.0	115
2003	0.0	0.0	1.0	1.0	1.0	0.0	1.0	0.0	1.0	0.5	1.0	0.9	0.0	1.0	1.0	0.0	1.0	187
2004	0.0	0.0	1.0	1.0	1.0	0.0	1.0	0.0	1.0	0.9	1.0	0.9	0.0	1.0	1.0	0.0	1.0	152
2005	0.0	0.0	1.0	1.0	1.0	0.0	1.0	0.0	0.9	1.0	1.0	1.0	0.0	1.0	1.0	0.0	1.0	154
2006	0.0	0.0	1.0	1.0	1.0	0.0	1.0	0.0	1.0	0.9	1.0	0.9	0.0	1.0	1.0	0.0	1.0	85
2007	0.0	0.0	1.0	1.0	1.0	0.1	1.0	0.0	1.0	0.9	1.0	0.9	0.0	1.0	1.0	0.0	1.0	232
2008	0.0	0.0	1.0	1.0	1.0	0.0	1.0	0.0	1.0	1.0	1.0	1.0	0.0	1.0	1.0	0.0	1.0	171
2009	0.0	0.0	1.0	1.0	1.0	0.0	1.0	0.0	1.0	1.0	1.0	0.5	0.0	1.0	1.0	0.0	1.0	301
2010	0.0	0.0	1.0	1.0	1.0	0.2	1.0	0.0	0.9	1.0	1.0	0.9	0.0	1.0	1.0	0.0	0.9	310
2011	0.0	0.0	1.0	1.0	1.0	0.3	1.0	0.0	0.9	1.0	1.0	0.9	0.0	1.0	1.0	0.0	1.0	452
2012	0.0	0.0	0.9	1.0	1.0	0.5	1.0	0.0	1.0	0.9	1.0	0.9	0.0	1.0	1.0	0.0	1.0	643

2013	0.0	0.0	0.9	1.0	1.0	0.9	1.0	0.0	1.0	0.5	1.0	1.0	0.0	1.0	1.0	0.0	1.0	517
2014	0.0	0.0	1.0	1.0	1.0	1.0	1.0	0.0	0.9	0.6	1.0	0.5	0.4	1.0	1.0	0.0	1.0	644
2015	0.0	0.0	1.0	1.0	1.0	1.0	1.0	0.0	0.9	0.9	1.0	0.2	0.7	1.0	1.0	0.0	1.0	877
2016	0.0	0.0	1.0	1.0	1.0	1.0	1.0	0.0	1.0	0.8	0.9	0.2	0.7	1.0	1.0	0.0	1.0	850
2017	0.0	0.0	1.0	1.0	1.0	1.0	1.0	0.0	0.9	0.9	0.7	0.0	0.9	1.0	1.0	0.0	1.0	1319
2018	0.0	0.0	1.0	1.0	1.0	1.0	1.0	0.0	1.0	0.8	0.8	0.0	0.8	1.0	1.0	0.0	1.0	857
2019	0.0	0.0	1.0	1.0	1.0	1.0	1.0	0.0	1.0	0.4	0.9	0.0	0.5	1.0	1.0	0.0	1.0	835
2020	0.0	0.0	1.0	1.0	1.0	1.0	1.0	0.0	0.9	0.3	0.4	0.1	0.8	1.0	1.0	0.0	1.0	93
2021	0.0	0.0	1.0	1.0	1.0	1.0	1.0	0.0	1.0	1.0	1.0	0.0	0.0	1.0	1.0	0.0	1.0	169
2022	0.0	0.0	1.0	1.0	1.0	1.0	1.0	0.0	0.9	1.0	0.9	0.0	0.0	1.0	1.0	0.0	1.0	1260

Supplementary Table 2. Hemagglutination inhibition assay using sera dilutions from ferrets vaccinated with H3 from 1968-2016.

Sera treated with turkey erythrocytes<br>**Start serum dilution is 1/20**<br>Hi in U-bottom and 20nM Oseltamivir (end conc.)						
			Passage	A/Bilth/16190/68	A/Neth/312/03	A/Sing/INFIMH-16-0019/2016
	HA	VD		F10051	F04001	F17042
A/Bilthoven/16190/1968	128	4	tMK5MDCK2 10-2	**2560**	<10	<10
NIB-104 Reass A/Singapore/INFIMH-16-0019/16	128	4	E6 11-Set-2017	<10	**10240**	<10
A/Neth/312/2003	128	4	xMDCK1 25-Jun-2010	<10	<10	**160**
Serum control				<10	<10	<10

Supplementary Table 3.

Cryo-EM data collection, refinement and validation statistics

	HA A/Hong kong/1/68 produced in GntI- cells<br><br>(EMDB-45997)<br>(PDB 9CXT)	EndoH-treated HA A/Hong kong/1/68<br><br>(EMDB-45998)<br>(PDB 9CXU)	HA A/Sing/INFIMH/16 produced in 293F cells<br><br>(EMDB-46500)<br>(PDB 9D2M)	HA A/Sing/INFIMH/16 produced in GntI- cells<br><br>(EMDB-46477)<br>(PDB 9D1U)	EndoH-treated HA A/Sing/INFIMH/16<br><br>(EMDB-46466)<br>(PDB 9D0Y)
**Data collection and processing**					
Microscope	Titan Krios	Arctica	Titan Krios	Arctica	Arctica
Magnification	29,000x	36,000x	29,000x	36,000	36,000
Voltage (kV)	300	200	300	200	200
Electron exposure (e–/Å^2)	50	50	50	50	50
Defocus range (μm)	-0.8 to -1.5	-0.8 to -1.5	-0.8 to -1.5	-0.8 to -1.5	-0.8 to -1.5
Detector	K2 Summit DED	Gatan K2 Summit	K2 Summit DED	Gatan K2 Summit	Gatan K2 Summit
Recording mode	Counting	Counting	Counting	Counting	Counting
Pixel size (Å)	1.026	1.15	1.026	1.15	1.15
Symmetry imposed	C3	C3	C3	C3	C3
Micrographs (no.)	497	1,141	1,714	631	1.064
Initial particle images (no.)	308,888	509,765	479,174	680,190	1,313,732
Final particle images (no.)	59,301	332,294	76,978	58,645	52,046
Map resolution (Å)	3.4	2.3	3.8	3.7	3.1
FSC threshold	0.143	0.143	0.143	0.143	0.143
Map sharpening *B* factor (Å^2)	-109.1	-67,0	-105.69	-141.4	-94.8
Map pixel size (Å)	1.026	1.15	1.026	1.15	1.15
Map resolution range (Å)	2.6-4.2	2.3-2.8	3.2-4.5	3.3-4.2	2.3-3.8
**Refinement**					
Initial model used (PDB code)	9CXT	9CXU	9D2M	9D1U	9D0Y
Model resolution (Å)	3.4	2.3	3.8	3.7	3.1
FSC threshold	0.5	0.5	0.5	0.5	0.5
Model resolution range (Å)	2.6-4.2	2.3-2.8	3.2-4.5	3.3-4.2	2.3-3.8
EMRinger score	2.36	5.49	3.31	3.82	4.56
Model composition					
Non-hydrogen	11,616	11,616	12,360	12,357	11,739
atoms	1,446	1,446	1,434	1,434	1,431
Protein residues	18	18	72	72	27

Ligands					
Mean *B* factors (Å^2)					
Protein	11.15	11.15	44.37	45.42	45.55
Ligand	33.94	33.94	61.55	96.64	70.67
R.m.s. deviations					
Bond lengths (Å)	0.021	0.021	0.023	0.023	0.023
Bond angles (°)	1.677	1.676	2.508	2.186	1.688
Validation					
MolProbity score	1.02	0.98	1.44	1.22	0.81
Clashscore	2.37	2.11	5.03	4.45	1.08
Poor rotamers (%)	0.00	0.00	0.00	0.00	0.00
Ramachandran plot					
Favored (%)	98.95	98.05	96.98	98.10	98.73
Allowed (%)	1.05	1.95	3.02	1.90	1.27
Disallowed (%)	0.00	0.00	0.00	0.00	0.00

References

1. NCBI Virus. https://www.ncbi.nlm.nih.gov/labs/virus/vssi/#/.
