# Supplementary material for: Structural and immunological characterization of the H3 influenza hemagglutinin during antigenic drift
Source: Nat Commun. 2025 Dec 11;16:11452. doi: 10.1038/s41467-025-66375-7 (PMC12749422; doi:10.1038/s41467-025-66375-7)
Supplement: Supplementary file 2 — Description of Additional Supplementary Files [file 41467_2025_66375_MOESM2_ESM.pdf]

Description of Additional Supplementary Files

File Name: Supplementary Data 1
Description: **Non-PNGS mutations on H3 HA circulating strains over time.** Analysis of the non-glycosylation related mutations on H3 HA circulating strains on the year interval when the glycan was added. The non-glycosylation related mutations on HA H3 circulating strains were determined after the analysis of ~11,000 sequences, which were downloaded from the influenza research database. The numbering corresponds to HK/68 reference sequence.
